# Skin Pigmentation: Is the Control of Melanogenesis a Target within Reach?

**DOI:** 10.3390/ijms19124040

**Published:** 2018-12-14

**Authors:** Alessandra Napolitano, Shosuke Ito

**Affiliations:** 1 Department of Chemical Sciences, University of Naples Federico II, Complesso MS Angelo, Via Cintia 4, I-80126 Naples, Italy; 2 Department of Chemistry, Fujita Health University School of Health Sciences, 1-98 Dengakugakubo, Kutsukake-cho, Toyoake, Aichi 470-1192, Japan

Skin pigmentation represents one of the most peculiar traits of human beings and its alteration as a consequence of pathological conditions has a dramatic impact on the wellness of individuals and their social relationships. Therefore, the possibility of controlling pigmentation by intervention at the different stages of the melanogenic process has represented over the years a most important research target, with many implications for the dermatological and cosmetic fields. One way of reaching such a goal is an in-depth knowledge of the genetics of pigmentation, the mechanism of activation of the melanocortin receptor 1 (MC1R) and the signaling cascade, and finally the expression and trafficking of melanogenic enzymes. In this regard, it is timely that Del Bino et al. [1] comprehensively summarized the current knowledge on biological and clinical aspects of skin pigmentation. 

Most of the papers and review articles appeared in this issue provide a contribution along two main lines, that is, a) unraveling hitherto unappreciated aspects of the complex melanogenesis machinery, or b) identifying new compounds or natural extracts with the ability to suppress or stimulate melanogenesis and elucidating their mechanism of action. 

Of particular interest is the review paper by Solano [2], which reported new data from recent studies on the nature of the metal cofactor of tyrosinase and tyrosinase-related proteins 1 and 2 (Trp-1 and Trp-2). Crystallographic analysis of recombinant proteins indicated that zinc ions rather than copper ions, as previously believed, act as cofactor of Trp-1 whose function is still controversial. To reconcile a series of conflicting observations, the author proposed that the enzyme could occur in two forms with different *N*-glycosylation occupancy, incorporating the two metal ions and acting on two different steps of melanogenesis, that is, Cu(II) to acquire 5,6-dihydroxyindole-2-carboxylic acid (DHICA) oxidase activity, or Zn(II) to acquire tautomerase activity. 

In Oh et al., *N*-acetyltransferase 10, so far considered as a valuable target for cancer and laminopathies, was shown to play a role also in melanogenesis and melanoma growth through regulation of microphthalmia-associated transcription factor (MITF) [3]. The evolutional aspects of MC1R and related genes were studied by Dib et al. [4]. The possibility of regulating melanogenesis by use of peptide analogs of α-MSH, the physiological agonist of the MC1R, whose activation triggers the melanogenic cascade in response also to UV exposure, was reviewed by Swope and Abdel-Malek [5]. In Campagne et al., 5-aminoacids peptides derived from adaptin 1 were shown to be able to decrease pigmentation by interfering with the interaction of the proteins that control maturation of melanosomes [6]. 

A series of compounds exhibiting a broad structural diversity have also been investigated for their antimelanogenic activity by much different mechanisms of action, ranging from direct inhibition of tyrosinase, as reported for thiazolyl resorcinols [7] or lipoylcaffeic ester conjugates [8], to MITF-mediated expression of melanogenic genes, as in the case of natural compounds (sesamol [9], zerumbone [10], protocatechuic acid from pear [11]), plant extracts (from *Oroxylum indicum* seeds) [12], Bamboo stems [13]), conditioned medium from stem cells [14], or synthetic derivatives (alkylglyceryl ascorbic acid derivatives [15], phenylhexa-1,3,5-trienes [16]). It should be emphasized that the study by Mann et al. [12] used human tyrosinase to perform structure-activity relationship, leading to the finding of highly potent tyrosinase inhibitors. Other processes in melanogenesis cascade are also targeted by natural sources (sesamol [16], *Polygonum tinctorium* flower extract [17]).

New strategies for melanogenesis stimulation are presented based on the use of low frequency [18] or pulsed electromagnetic fields [19] that induce an increase of the activity and expression of tyrosinase and Trp-2. Novel furocoumarin derivatives were shown to stimulate melanogenesis via up-regulation of MITF and TYR family [20]. 

The case of rhododendrol, a skin-whitening ingredient that was reported to induce leukoderma, was reviewed by Ito and Wakamatsu [21]. This provides an example of how a full understanding of the cytotoxicity of this compound was gained through detailed characterization of the tyrosinase-induced oxidation pathway with identification of quinonoid species capable of targeting SH cysteine residue of proteins as well as by assessment of sustained production of reactive oxygen species capable of inducing oxidative stress conditions in melanocytes. 

Finally, the opportunities opened by knowledge of the different chemical reactivity of the two major indole intermediates of melanogenesis, 5,6-dihydroxyindole (DHI) and DHICA, for cosmetic purposes were illustrated through examples in the field of hair dyeing and photoprotection [22]. A unique aspect of the roles of melanin was presented by Sarna et al. [23] showing that melanin is the dominating factor responsible for the mechanical properties of melanoma cells. Negro et al. reviewed evolutional aspect of pigmentation with special emphasis on mechanisms of color evolution by natural selection [24].

In conclusion, the research contributions presented in this issue clearly illustrate how investigation of the basic facets of the melanogenetic process and insight into the mechanism of action of natural and synthetic compounds may offer a valuable tool for the design of efficient and selective strategies for the control of hyperpigmentary disorders or melanin stimulation as required in vitiligo. In this regard, the current status of clinical and molecular aspects of vitiligo treatments is discussed by Bishnoi and Parsad [25].
